# Orexin B Reduces Cerebral Aneurysms Through Inhibition of SP‐1

**DOI:** 10.1002/cns.70958

**Published:** 2026-06-08

**Authors:** Lei Chen, Jinlong Xu, Fengyun Ye, Jiajia Luo, Huimin Yu

**Affiliations:** ^1^ Department of Neurosurgery The First Dongguan Affiliated Hospital, Guangdong Medical University Dongguan Guangdong China; ^2^ Department of Neurology The First Dongguan Affiliated Hospital, Guangdong Medical University Dongguan Guangdong China

**Keywords:** cerebral aneurysms, human brain microvascular endothelial cells, macrophage, orexin B, SP‐1

## Abstract

**Background:**

Cerebral aneurysms (CAs) are pathological dilations of intracranial arteries with high rupture risk, yet the molecular mechanisms driving their formation remain incompletely understood. The Orexin B/OX2R system, known for regulating arousal and metabolism, has recently been implicated in vascular pathology, but its role in CAs has not been explored.

**Methods:**

Serum Orexin A and B levels were measured in 38 CA patients and 43 healthy controls. A murine CA model (elastase‐induced) was established using wild‐type (WT) and OX2R knockout (*OX2R* ^−/−^) mice, with or without Orexin B treatment (30 μg/kg/day for 7 weeks). Aneurysm size, inflammatory mediators (IL‐6, MMP‐9, MCP‐1, E‐selectin), macrophage infiltration (CD68), and SP‐1 expression were assessed. In vitro studies using human brain microvascular endothelial cells (HBMVECs) examined Ang II‐induced OX2R suppression, monocyte adhesion, and SP‐1‐mediated signaling following Orexin B treatment and SP‐1 overexpression.

**Results:**

CA patients and mice exhibited significantly reduced serum Orexin B levels (CA patients: 3.21 ± 0.52 vs. controls: 8.56 ± 1.23 pg/mL, *p* < 0.01), with no change in Orexin A. OX2R expression was downregulated in the circle of Willis of CA mice. Orexin B administration attenuated aneurysm formation in WT mice (size reduction from 3.72 ± 0.469 mm to 1.93 ± 0.252 mm, *p* < 0.01) but not in *OX2R* ^−/−^ mice. Orexin B suppressed IL‐6, MMP‐9, MCP‐1, and E‐selectin expression, reduced CD68+ macrophage infiltration, and decreased SP‐1 levels in WT but not *OX2R* ^−/−^ mice. In HBMVECs, Ang II dose‐dependently reduced OX2R expression. Orexin B inhibited Ang II‐induced monocyte adhesion and SP‐1‐mediated pro‐inflammatory signaling, effects abolished by OX2R siRNA or SP‐1 overexpression.

**Conclusions:**

The Orexin B/OX2R axis is dysregulated in CAs, and Orexin B protects against CA formation through OX2R‐dependent anti‐inflammatory mechanisms involving SP‐1 suppression. These findings identify the Orexin B/OX2R/SP‐1 pathway as a potential therapeutic target for cerebral aneurysms.

## Graphical abstract

Orexin B/OX2R signaling attenuates cerebral aneurysm formation by suppressing SP‐1‐mediated inflammation, macrophage infiltration, and endothelial adhesion, with OX2R knockdown abolishing these protective effects. The study highlights Orexin B as a therapeutic candidate targeting vascular inflammation and remodeling in aneurysms.

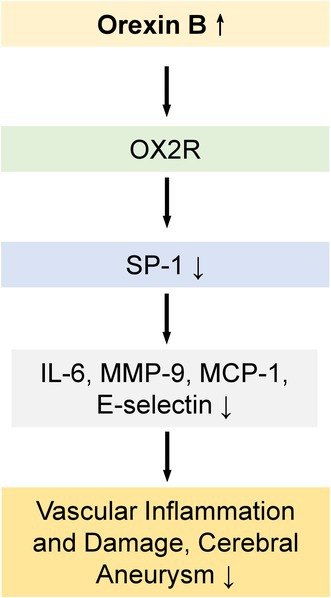

## 1 Introduction

Cerebral aneurysms (CAs) are focal dilations of intracranial arteries that pose a significant public health risk due to their potential rupture, which can result in subarachnoid hemorrhage with high associated morbidity and mortality [1, 2]. Although intracranial aneurysms are present in an estimated 3%–5% of the general population [3], the precise mechanisms governing their formation, progression, and rupture remain incompletely elucidated.

CA pathogenesis is multifactorial, involving genetic predisposition, hemodynamic stress, and inflammatory processes [4]. Hemodynamic forces, such as wall shear stress, contribute to arterial wall injury and initiate aneurysm formation [4]. Concurrently, inflammation plays a critical role, characterized by macrophage infiltration into the vessel wall and the release of cytokines and matrix metalloproteinases (MMPs) that degrade the extracellular matrix and weaken vascular integrity [5].

The Orexin system, comprising Orexin A and B peptides and their receptors OX1R and OX2R, is well‐established in regulating wakefulness and energy homeostasis [6]. Emerging evidence also implicates this system in vascular function [7]. While Orexin A has documented vasoconstrictive properties [8], the role of Orexin B in vascular physiology, particularly in the context of CA, is largely unexplored.

A few previous studies have hinted at a potential link between the Orexin system and vascular diseases. For example, alterations in the expression of Orexin receptors have been observed in some cardiovascular disorders [9]. Moreover, in a pilot study, cerebrospinal fluid orexin levels were found to be associated with the consciousness level in patients with aneurysmal subarachnoid hemorrhage, suggesting a possible role of orexin in the pathophysiology of such conditions [10]. Given the importance of maintaining vascular integrity in preventing CAs and the limited understanding of the role of the Orexin B/OX2R axis in cerebral vasculature, we hypothesized that dysregulation of this system might be involved in the pathogenesis of CAs.

The Orexin B/OX2R axis could potentially influence CA development through multiple mechanisms. Orexin B is an endogenous neuropeptide agonist for the G‐protein‐coupled OX2R [11]. In the nervous system, Orexin B‐mediated activation of OX2R has been associated with the regulation of wakefulness and arousal [12]. However, its effects on the vascular system, particularly in the context of CAs, are yet to be explored. SP‐1's role in CA pathogenesis represents an emerging focus, as this transcription factor controls inflammatory and extracellular matrix‐related genes implicated in vascular destabilization [13]. Orexins additionally intersect with stroke‐related neuroinflammation and cognitive deficits, processes sharing overlapping mechanisms with CA pathophysiology [14]. Their therapeutic potential in immune‐mediated disorders underscores their relevance to CA development [15].

To date, few studies have fully explored Orexin B's role in CAs. Our study addresses this gap by analyzing Orexin B and OX2R expression in human CA tissues and experimental models, while probing Orexin B's influence on aneurysm formation and linked molecular mechanisms. Elucidating this pathway's impact may reveal actionable therapeutic targets to disrupt CA progression.

## 2 Materials and Methods

### 2.1 Human Samples

Ethical oversight for this research was managed by the Medical Ethics Committee at the First Dongguan Affiliated Hospital at Guangdong Medical University (Approval No. NTZ5686). A written agreement was obtained from every individual before their participation. Blood collection and data handling followed ethical standards set by the Declaration of Helsinki. 38 CA patients and 43 matched controls (healthy volunteers from routine health check‐ups) were enrolled from affiliated hospital clinics and wards. Controls were matched to patients for age (±5 years) and sex, and were confirmed to have no acute infections, autoimmune conditions, or significant vascular comorbidities. CA diagnosis was confirmed via DSA or CTA, excluding individuals with acute infections, autoimmune conditions, or significant vascular comorbidities. Peripheral blood (5 mL) was drawn into EDTA tubes, centrifuged (3000 rpm, 10 min, 4°C), and serum aliquots were preserved at −80°C for subsequent analysis.

### 2.2 Mouse Samples

Procedures involving animals were reviewed and authorized by the Animal Ethics Oversight Board of the First Dongguan Affiliated Hospital at Guangdong Medical University (Approval No. NTC5701). Work adhered to ARRIVE recommendations and local laws governing humane animal research. Housing conditions prioritized welfare, with routine checks by ethics staff to ensure compliance. Eight‐week‐old male C57BL/6 WT (GemPharmatech, #C001122) and *OX2R* ^−/−^ mice (GemPharmatech, #T003467) were used (*n* = 60). The CA model was induced via elastase perfusion of the isolated right common carotid artery as described [16, 17, 18]. Briefly, the right common carotid artery was isolated, temporary ligatures were placed, and a 0.1 mL solution of type I porcine pancreatic elastase (10 U/mL in saline; Sigma E1250) was infused into the isolated segment for 20 min. Controls underwent saline perfusion. After 7 weeks, blood was collected via cardiac puncture, and the COW region was dissected—half snap‐frozen for molecular analysis, half fixed in 4% PFA for histology. For Orexin B treatment, mice received daily intraperitoneal injections of Orexin B (30 μg/kg in PBS; Phoenix Pharmaceuticals, #003‐32) or an equal volume of PBS (vehicle control), starting 24 h post‐surgery and continuing for 7 weeks.

### 2.3 Serum and CSF Orexin Measurement

Orexin A/B levels were quantified using ELISA kits (Phoenix Pharmaceuticals: EK‐060‐12/Orexin A, EK‐061‐12/Orexin B). Serum/CSF/standards (100 μL) were incubated (2 h, RT), followed by sequential incubations with biotin‐antibody (1 h), streptavidin‐HRP (30 min), and substrate (15 min, dark). Absorbance (450 nm) was measured post‐stop (BioTek microplate reader).

### 2.4 qRT‐PCR Analysis

RNA was isolated from COW tissue and HBMVECs using TRIzol (Invitrogen 15,596,026), with concentration/purity verified by NanoDrop 2000. cDNA was synthesized from 1 μg RNA (High‐Capacity cDNA Kit, Applied Biosystems 4,368,814). qPCR was performed on a QuantStudio 5 system (PowerUp SYBR Green Master Mix, Applied Biosystems A25742) with PrimerBank‐derived primers. Sequences (listed 5‐prime to 3‐prime) were: Mouse *OX2R* F: CCCTCTCTCGTCGCAACTG, R: GTCGTCATAGTCCGTGGGA; Mouse *IL‐6* F: TAGTCCTTCCTACCCCAATTTCC, R: TTGGTCCTTAGCCACTCCTTC; Mouse *Mmp9* F: CTGGACAGCCAGACACTAAAG, R: CTCGCGGCAAGTCTTCAGAG; Mouse *Mcp1* F: GAGGACAGATGTGGTGGGTTT, R: AGGAGTCAACTCAGCTTTCTCTT; Mouse *E‐selectin* F: ATGCCTCGCGCTTTCTCTC, R: GTAGTCCCGCTGACAGTATGC; Mouse *Sp1* F: GCCGCCTTTTCTCAGACTC, R: TTGGGTGACTCAATTCTGCTG; Mouse *Gapdh* F: AGGTCGGTGTGAACGGATTTG, R: TGTAGACCATGTAGTTGAGGTCA; Human *SP‐1* F: AGTTCCAGACCGTTGATGGG, R: GTTTGCACCTGGTATGATCTGT; Human *GAPDH* F: CTGGGCTACACTGAGCACC, R: AAGTGGTCGTTGAGGGCAATG. Cycling conditions: 95°C for 10 min; 40 cycles of 95°C (15 s) and 60°C (1 min). Gene expression was normalized to GAPDH via the 2^−ΔΔCT^ method.

### 2.5 Western Blot Analysis

Proteins were extracted from COW tissue and HBMVECs using RIPA buffer (Thermo Fisher 89,900) supplemented with protease/phosphatase inhibitors (Thermo Fisher 78,441). Protein concentrations were quantified via BCA assay (Thermo Fisher 23,227). Samples (30 μg) were resolved by 10% SDS‐PAGE and transferred to PVDF membranes (Millipore IPVH00010). After blocking with 5% non‐fat milk/TBST (1 h, RT), membranes were probed overnight at 4°C with primary antibodies: anti‐OX2R (Abcam ab183072; 1:1000), anti‐IL‐6 (Cell Signaling 12,912; 1:1000), anti‐MMP‐9 (Abcam ab38898; 1:1000), anti‐MCP‐1 (Abcam ab25124; 1:1000), anti‐E‐selectin (Thermo Fisher 14–0627‐82; 1:1000), anti‐SP‐1 (Cell Signaling 13,370; 1:1000), and anti‐β‐actin (Cell Signaling 8457; 1:5000) for cell lysates, as appropriate. Following TBST washes, HRP‐conjugated secondary antibodies (Jackson ImmunoResearch; 1:5000) were applied (1 h, RT). Signals were detected using ECL (Thermo Fisher 32,109) on a ChemiDoc XRS+ system (Bio‐Rad) and analyzed with Image Lab software (GAPDH‐normalized).

### 2.6 Histological Analysis

COW samples fixed in 4% paraformaldehyde were paraffin‐embedded and sectioned (5 μm). Elastic fibers were visualized using Verhoeff‐van Gieson staining, with aneurysm size measured as maximal sac diameter (ImageJ). For IHC, sections underwent antigen retrieval (citrate buffer, pH 6.0, 95°C, 20 min), peroxidase blocking (3% H_2_O_2_, 10 min), and incubation with anti‐CD68 (Abcam ab955; 1:200, 4°C overnight). After PBS washes, HRP‐conjugated secondary antibody was applied (1 h, RT), followed by DAB development and hematoxylin counterstaining. CD68+ macrophages were quantified in five random (×400) fields per section. For immunofluorescence, sections were stained with anti‐OX2R (Abcam ab183072) and anti‐CD31 (Abcam ab28364), followed by appropriate secondary antibodies and DAPI counterstaining.

### 2.7 Cell Culture and Treatments

Human brain microvascular endothelial cells (HBMVECs; Procell Life Science & Technology Co. Ltd., Wuhan, China) were cultured in endothelial cell medium (ECM; Procell; catalog number 1101) with 5% fetal bovine serum (FBS; Gibco, Thermo Fisher Scientific), 1% endothelial cell growth supplement (ECGS; Procell), and 1% penicillin–streptomycin (Gibco) at 37°C in 5% CO_2_ humidified air. Cells were passaged every 3–4 days. For Ang II treatment, HBMVECs were serum‐starved for 12 h, then treated with Ang II (Sigma‐Aldrich; catalog number A9525) at 50 nM and 100 nM for 24 h. For Orexin B treatment, HBMVECs were pre‐treated with Orexin B (Phoenix Pharmaceuticals; catalog number B010301) at 50 nM and 100 nM for 30 min before Ang II treatment. For siRNA knockdown, HBMVECs were transfected with 50 nM OX2R siRNA (Santa Cruz sc‐42,956) or scramble control using Lipofectamine RNAiMAX, then treated as indicated.

### 2.8 Monocyte Adhesion Assay

THP‐1 human monocytic cells (ATCC, Manassas, VA, USA; catalog number TIB‐202) were labeled with Calcein‐AM (Invitrogen; catalog number C3100MP; 5 μM) for 30 min at 37°C. After washing, 1 × 10^5^ labeled THP‐1 cells were added to each well of a 24‐well plate with confluent HBMVECs and incubated for 1 h at 37°C. Non‐adherent cells were removed by gentle 3‐time PBS washes. Adherent cell number was determined by measuring fluorescence intensity (excitation 485 nm, emission 530 nm) using a microplate reader (BioTek Instruments).

### 2.9 Lentiviral Vector Transduction

To overexpress SP‐1 in HBMVECs, lentiviral vectors with the *SP‐1* gene (GeneCopoeia, #EX‐Z4922‐Lv105) and a control vector (GeneCopoeia, #EX‐NEG‐Lv105) were used. HBMVECs were seeded in 6‐well plates at 5 × 10^4^ cells/well. At 70%–80% confluence, cells were transduced with lentiviral vectors at an MOI of 10 with polybrene (8 μg/mL; Sigma‐Aldrich). After 24 h, the medium was refreshed. Stable transfectants were selected with puromycin (2 μg/mL; Sigma‐Aldrich) for 7 days.

### 2.10 Statistical Analysis

Statistical analysis results are expressed as mean ± SD. All statistical evaluations were conducted in GraphPad Prism (GraphPad Software). Intergroup comparisons utilized unpaired Student's *t‐*tests, while multigroup analyses employed one‐way or two‐way ANOVA with Tukey's multiple comparisons test. Statistical significance was established at *p* < 0.05. All bar graphs include superimposed scatter plots showing individual data points.

## 3 Results

### 3.1 Serum Orexin B Levels Are Reduced in CA Patients

**FIGURE 1:**
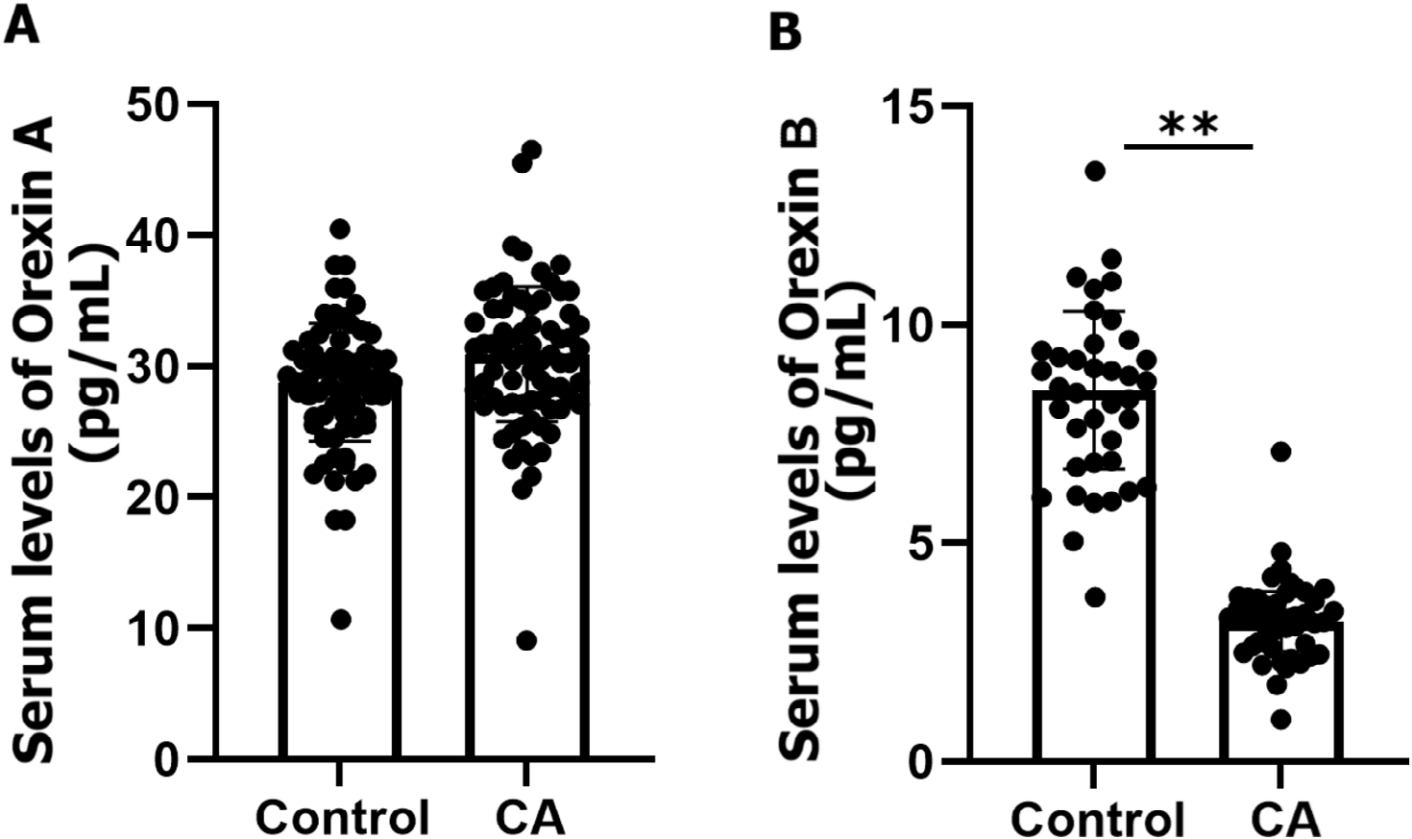
Serum levels of Orexin A and Orexin B in normal individuals and CA patients. (A) Serum Orexin A levels in normal subjects (29.5 ± 4.21 pg/mL) and CA patients (31.7 ± 5.33 pg/mL) showed no significant difference (*p* > 0.05). (B) Serum Orexin B levels were significantly reduced in CA patients (3.21 ± 0.52 pg/mL) versus normal controls (8.56 ± 1.23 pg/mL; **, *p* < 0.01), suggesting Orexin B's involvement in CA pathophysiology. Data are mean ± SD; scatter plots show individual values. Unpaired two‐tailed *t*‐test. *n* = 38 patients, 43 controls.

CAs are complex vascular disorders with multifactorial causes. To explore the Orexin B/OX2R system's role in CA pathogenesis, we measured serum Orexin A and B levels in normal individuals and CA patients. As Figure 1A showed, there was no significant difference in serum Orexin A levels between normal subjects (29.5 ± 4.21 pg/mL) and CA patients (31.7 ± 5.33 pg/mL, *p* > 0.05). Conversely, Figure 1B showed a significant drop in serum Orexin B levels in CA patients (3.21 ± 0.52 pg/mL) compared to controls (8.56 ± 1.23 pg/mL, *p* < 0.05). These findings imply that Orexin B, not Orexin A, may be involved in CA pathophysiology.

### 3.2 Dysregulation of the Orexin B/OX2R System in a Mouse Model of CA

We established a mouse CA model to further study the Orexin B/OX2R system. Representative H&E images illustrate the key pathological changes in the aneurysm dome of CA mice relative to controls: a significant decrease in vascular wall layering and smooth muscle cell abundance, accompanied by elastic fiber fragmentation and intimal destruction (Figure S1).

**FIGURE 2:**
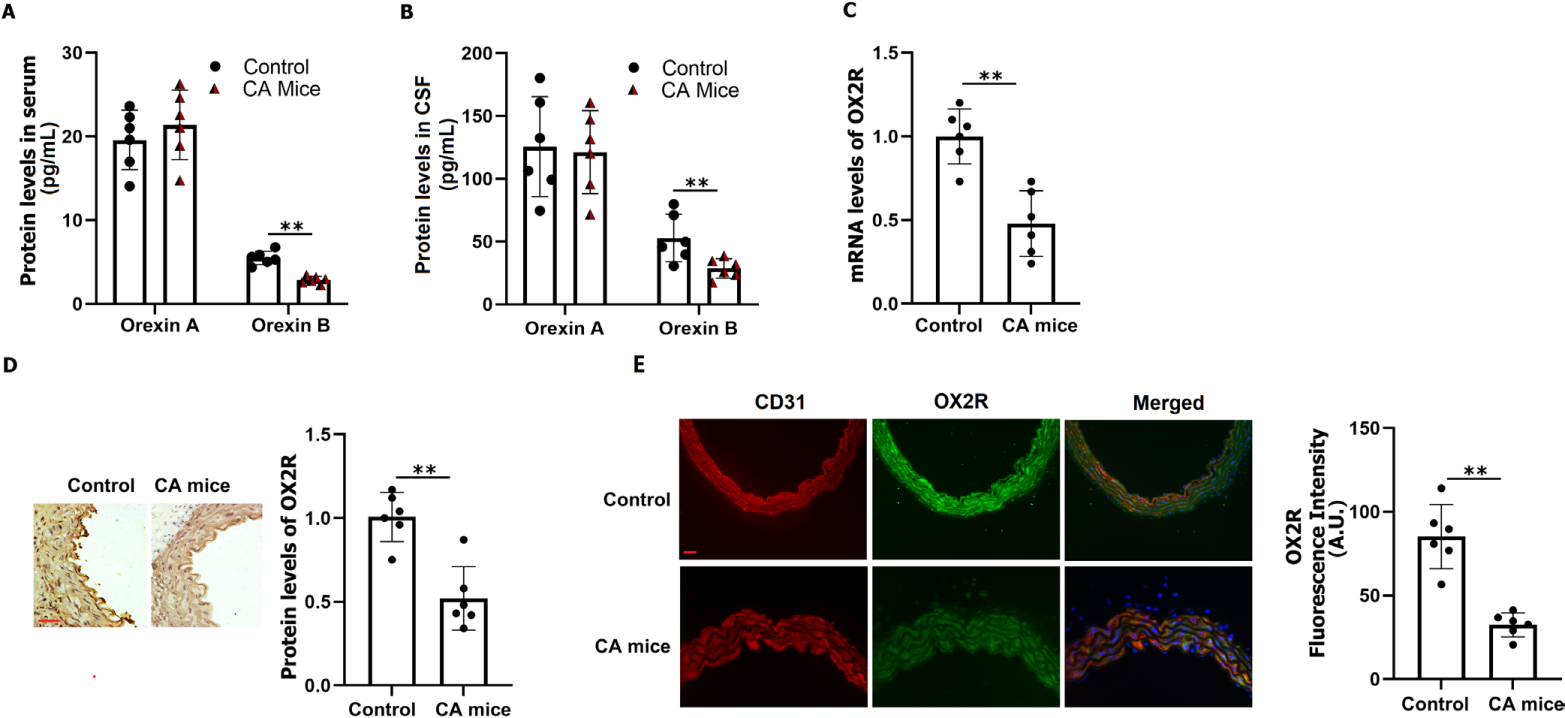
Dysregulation of the Orexin B/OX2R system in a mouse model of CA. Serum Orexin A (control: 19.6 ± 2.57 pg/mL; CA mice: 21.4 ± 2.86 pg/mL, *p* > 0.05) and Orexin B (control: 5.39 ± 0.78 pg/mL; CA mice: 2.88 ± 0.45 pg/mL, **p < 0.01) levels, with a significant decrease in Orexin B in CA mice. (B) CSF Orexin A (control: 125.6 ± 18.21 pg/mL; CA mice: 121.3 ± 16.59 pg/mL, *p* > 0.05) and Orexin B (control: 52.8 ± 7.05 pg/mL; CA mice: 28.6 ± 3.08 pg/mL, **, *p* < 0.01) levels, with a significant decrease in Orexin B in CA mice. (C) qRT‐PCR revealed downregulated *OX2R* mRNA in the circle of Willis (COW) region of CA mice. (D) Immunohistochemistry confirmed reduced OX2R protein levels in the COW region of CA mice. (E) Representative immunofluorescence images showing colocalization of OX2R (green) with the endothelial marker CD31 (red) in the COW region of control and CA mice. Scale bar, 50 μm. Data are mean ± SD; scatter plots show individual values. *n* = 6 mice/group. **, *p* < 0.01 vs. control group (unpaired two‐tailed *t*‐test).

As shown in Figure 2A, like in humans, serum Orexin A levels didn't differ significantly between control mice (19.6 ± 2.57 pg/mL) and CA mice (21.4 ± 2.86 pg/mL, *p* > 0.05). However, serum Orexin B levels were notably lower in CA mice (2.88 ± 0.45 pg/mL) than in controls (5.39 ± 0.78 pg/mL, *p* < 0.01). There was no statistically significant difference in cerebrospinal fluid (CSF) Orexin A levels between control mice (125.6 ± 18.21 pg/mL) and CA mice (121.3 ± 16.59 pg/mL, *p* > 0.05). In contrast, CSF Orexin B levels were significantly reduced in CA mice (28.6 ± 3.08 pg/mL) compared to controls (Figure 2B, 52.8 ± 7.05 pg/mL, *p* < 0.01).

For OX2R expression, Figure 2C,D showed that OX2R was downregulated in the circle of Willis (COW) region of CA mice. Quantitative real‐time PCR revealed lower *OX2R* mRNA levels in CA mice than in controls (Figure 2C: 0.45 ± 0.05 vs. 1 ± 0.11). Immunohistochemistry analysis confirmed the decrease in OX2R protein levels in the COW region of CA mice (Figure 2D: 0.53 ± 0.05 vs. 1 ± 0.14). Immunofluorescence co‐staining for OX2R and the endothelial marker CD31 confirmed OX2R expression in endothelial cells of the COW region, which was markedly diminished in CA mice (Figure 2E). These results suggest the Orexin B/OX2R system is dysregulated in the CA mouse model.

### 3.3 Orexin B Administration Attenuates CA Formation in Wild‐Type but Not OX2R Knockout Mice

**FIGURE 3:**
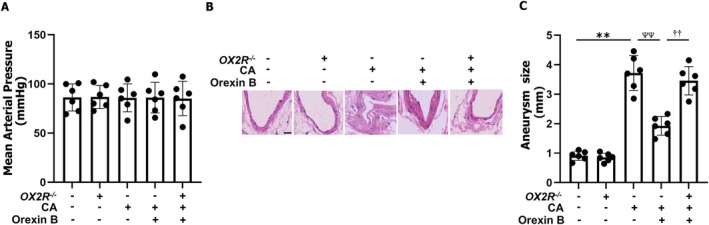
Administration of Orexin B prevents CA formation in wild‐type but not OX2R knockout mice. (A) Systolic blood pressure (SBP) and mean arterial pressure (MAP) at 7 weeks post‐surgery showed no significant differences across groups. (B) Representative Verhoeff‐van Gieson staining images showed reduced aneurysm formation in CA + Orexin B wild‐type mice. Scale bars: 50 μm. (C) Aneurysm size was significantly smaller in CA + Orexin B mice (1.93 ± 0.252 mm) versus CA mice (3.72 ± 0.469 mm; *p* < 0.05), with no effect in *OX2R* ^−/−^ mice. Data are mean ± SD; scatter plots show individual values. *n* = 6 mice/group. **, *p* < 0.01 vs. WT group; ΨΨ, *p* < 0.01 vs. CA group; ††, *p* < 0.01 vs. CA + Orexin B group (two‐way ANOVA with Tukey's test).

To determine the functional role of Orexin B in CA development, we administered Orexin B to wild‐type (WT) and OX2R knockout (*OX2R* ^−/−^) mice with induced CAs. Systolic and mean arterial pressure (MAP) were measured non‐invasively at 7 weeks post‐surgery. As shown in Figure 3A, there were no significant differences in systolic blood pressure among the different groups of mice at 7 weeks after surgery, ruling out the confounding effect of blood pressure on CA formation. Figure 3B presents representative images of Verhoeff‐van Gieson staining of the vascular wall, which clearly showed the reduced aneurysm formation in WT mice treated with Orexin B. As shown in Figure 3C, the quantification of aneurysm size offered key insights into the effect of Orexin B on CA formation. In the CA group, the average aneurysm size was 3.72 ± 0.469 mm. In wild‐type (WT) mice with CAs treated with Orexin B (CA + Orexin B group), the size significantly decreased to 1.93 ± 0.252 mm (*p* < 0.01). In OX2R knockout (*OX2R* ^−/−^) mice with CAs, the aneurysm size was 3.46 ± 0.485 mm. Treatment with Orexin B in these mice (CA + *OX2R* ^−/−^ + Orexin B group) did not change the size. Compared to the CA group, there was no significant reduction. This indicates that without the OX2R receptor, Orexin B cannot protect against CA formation, suggesting its effect is mediated through OX2R.

### 3.4 Orexin B Inhibits the Expression of Inflammatory Mediators in the COW Region

**FIGURE 4:**
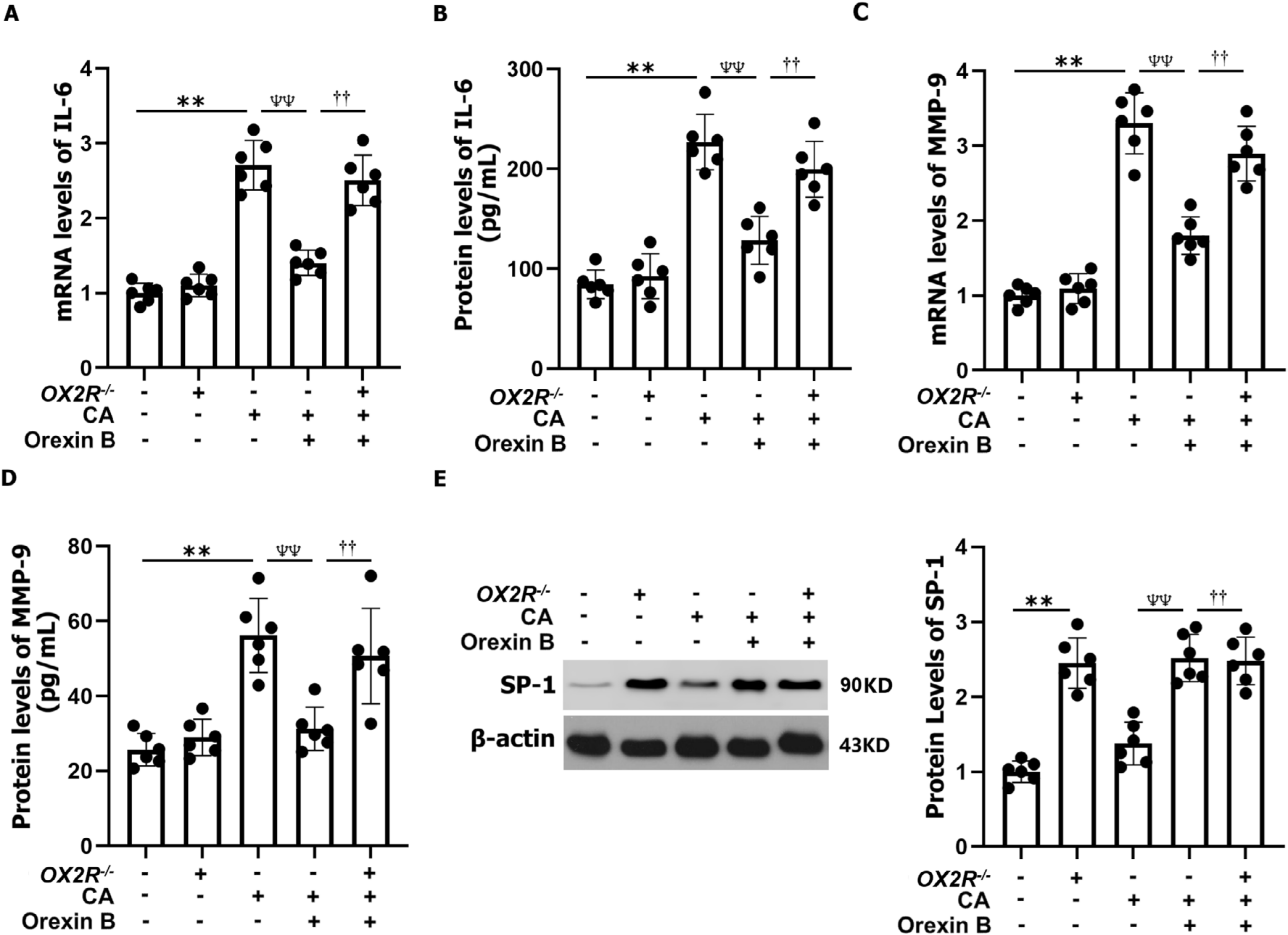
Administration of Orexin B inhibits inflammatory mediator expression in the COW region. (A) *IL‐6* mRNA levels in the COW region were elevated in the CA group (2.7 ± 0.29) versus WT (1 ± 0.09) and reduced by Orexin B (CA + Orexin B: 1.4 ± 0.13). (B) ELISA‐measured IL‐6 protein levels mirrored this trend. (C) *MMP‐9* mRNA levels increased in CA mice (3.3 ± 0.32) and decreased with Orexin B (1.8 ± 0.22). (D) MMP‐9 protein levels showed similar reductions in WT mice, absent in *OX2R* ^−/−^ mice. (E) Western blot analysis showing SP‐1 protein levels in the COW region. Data are mean ± SD; scatter plots show individual values. *n* = 6 mice/group. **, *p* < 0.01 vs. WT group; ΨΨ, *p* < 0.01 vs. CA group; ††, *p* < 0.01 vs. CA + Orexin B group (two‐way ANOVA with Tukey's test).

Inflammation is a crucial factor in CA pathogenesis. All measurements were performed at the 7‐week endpoint. We examined the effect of Orexin B on the expression of key inflammatory mediators, interleukin‐6 (IL‐6) and matrix metalloproteinase‐9 (MMP‐9), in the COW region of mice. Figure 4A demonstrated that the mRNA levels of *IL‐6* were significantly increased in the CA group (2.7 ± 0.29) compared to the WT group (1 ± 0.09). Treatment with Orexin B in WT mice (CA + Orexin B group) reduced the mRNA levels of *IL‐6* to 1.4 ± 0.13. Similarly, ELISA analysis in Figure 4B demonstrated a significant increase in IL‐6 protein levels in the CA group (227.3 ± 32.51 pg/mL) compared to the WT group (84.5 ± 11.37 pg/mL), and Orexin B treatment decreased the protein levels to 128.5 ± 18.33 pg/mL. For *MMP‐9*, Figure 4C showed that the mRNA levels were elevated in the CA group (3.3 ± 0.32) compared to the WT group (1 ± 0.13), and Orexin B treatment in WT mice (CA + Orexin B group) decreased the mRNA levels to 1.8 ± 0.22. Figure 4D demonstrated the corresponding protein levels, with a significant increase in the CA group (56.2 ± 7.36) and a reduction in the CA + Orexin B group (31.5 ± 3.53). In *OX2R* ^−/−^ mice, the inhibitory effect of Orexin B on the expression of IL‐6 and MMP‐9 was abolished. Western blot analysis also revealed increased SP‐1 protein in the COW of CA mice, which was normalized by Orexin B treatment in a manner dependent on OX2R (Figure 4E). These results suggest that the anti‐inflammatory effect of Orexin B is OX2R‐dependent.

### 3.5 Orexin B Inhibits Macrophage Infiltration and the Expression of Chemokine and Adhesion Molecules

**FIGURE 5:**
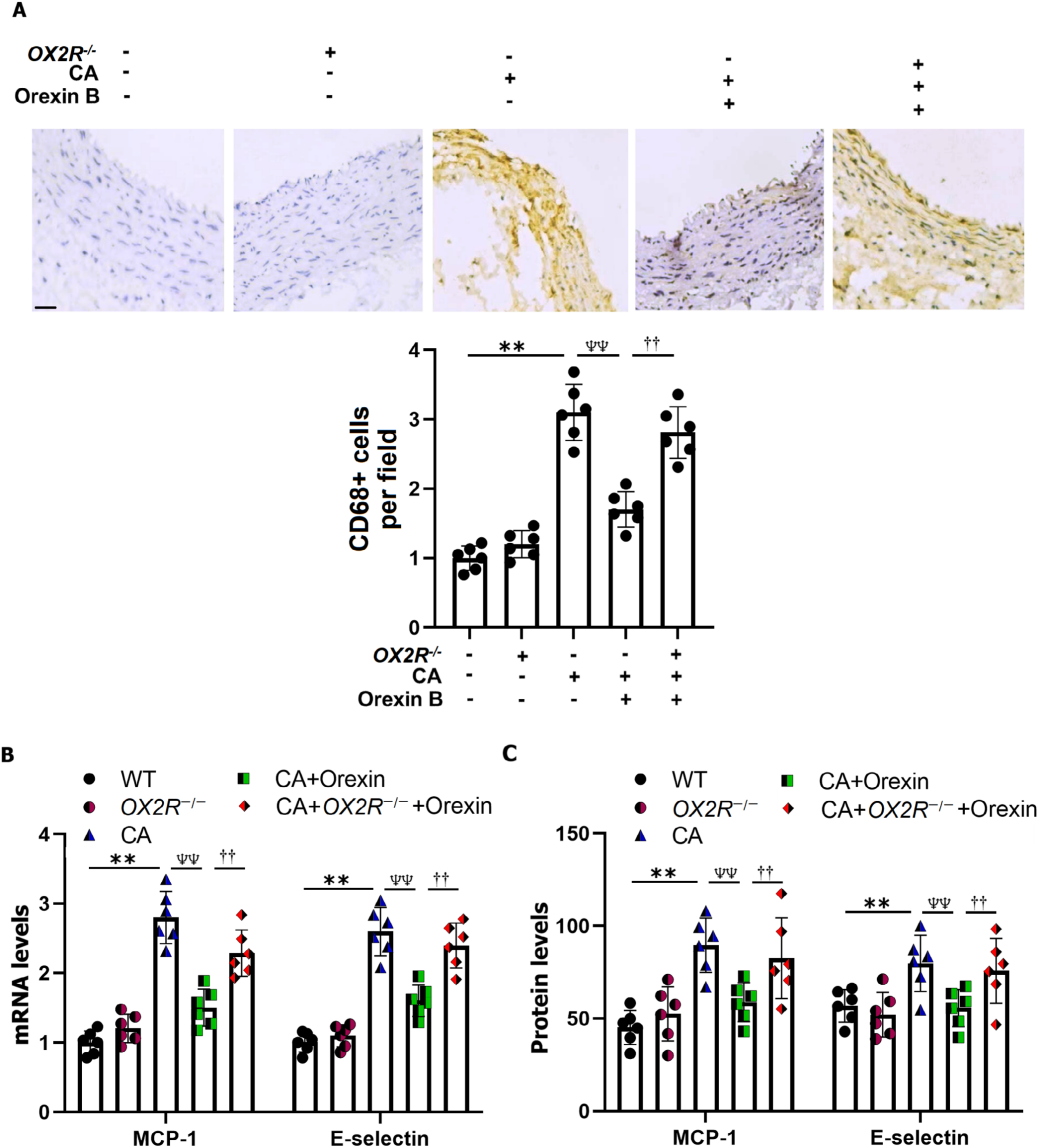
Orexin B inhibits macrophage infiltration and adhesion molecule expression. (A) CD68 staining showed increased macrophages in CA mice (3.1 ± 0.35) versus WT (1 ± 0.24), reduced by Orexin B in WT (CA + Orexin B: 1.7 ± 0.21). Representative IHC images for CD68 (brown) are shown. Scale bar, 50 μm. (B) mRNA levels of *MCP‐1* and *E‐selectin* were elevated in CA mice and decreased by Orexin B. (C) ELISA‐measured protein levels followed the same pattern, with no inhibitory effect in *OX2R* ^−/−^ mice. Data are mean ± SD; scatter plots show individual values. *n* = 6 mice/group. **, *p* < 0.01 vs. WT group; ΨΨ, *p* < 0.01 vs. CA group; ††, *p* < 0.01 vs. CA + Orexin B group (two‐way ANOVA with Tukey's test).

Macrophage infiltration and the expression of chemokines and adhesion molecules play important roles in the inflammatory process of CAs. All measurements were performed at the 7‐week endpoint. Representative CD68 IHC images are shown in Figure 5A. Results demonstrate that the number of CD68‐positive macrophages was significantly increased in the CA group (3.1 ± 0.35) compared to the WT group (1 ± 0.24). Treatment with Orexin B in WT mice (CA + Orexin B group) reduced the number of macrophages to 1.7 ± 0.21. Figure 5B displays the mRNA levels of the chemokine *MCP‐1* and the endothelial adhesion molecule *E‐selectin* in the COW region. The mRNA levels of both *MCP‐1* and *E‐selectin* were increased in the CA group compared to the WT group, and Orexin B treatment in WT mice decreased their expression. ELISA in Figure 5C further confirmed the increase in MCP‐1 and E‐selectin protein levels in the CA group and the reduction after Orexin B treatment in WT mice. Similar to the results of inflammatory mediators, the inhibitory effect of Orexin B on macrophage infiltration and the expressions of MCP‐1 and E‐selectin were not observed in *OX2R* ^−/−^ mice. *OX2R* ^−/−^ mice exhibited no significant differences in baseline or CA‐induced levels of IL‐6 and MMP‐9 compared to WT mice (Figure S2), indicating that the knockout primarily affects the response to Orexin B.

### 3.6 Ang II Reduces the Expression of OX2R in Human Brain Microvascular Endothelial Cells

We aimed to uncover the mechanism behind the downregulation of OX2R in.

**FIGURE 6:**
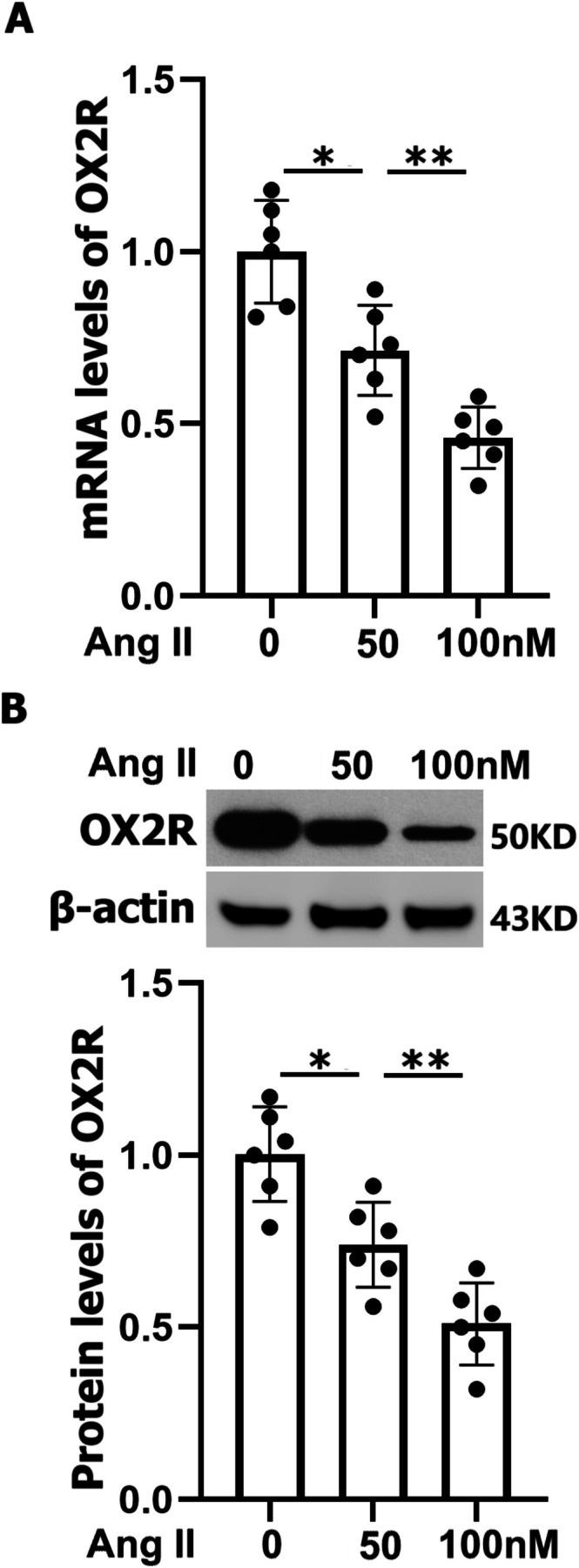
Ang II reduces OX2R expression in HBMVECs. (A) Real‐time PCR showed dose‐dependent decreases in *OX2R* mRNA in HBMVECs treated with Ang II. (B) Western blot analysis confirmed dose‐dependent reductions in OX2R protein levels. *n* = 6/group. *, **, *p* < 0.05, 0.01 vs. Control group (one‐way ANOVA with Tukey's test).

CAs. Given that angiotensin II (Ang II) is well‐known as a factor influencing vascular remodeling, we studied its effect on OX2R expression in human brain microvascular endothelial cells (HBMVECs). Figure 6A showed that when HBMVECs were treated with 50 nM and 100 nM of Ang II for 24 h, there was a clear dose‐dependent drop in *OX2R* mRNA levels. The mRNA levels were 0.71 ± 0.075 and 0.46 ± 0.043 in the groups treated with 50 and 100 nM Ang II, respectively, while the control group had a level of 1 ± 0.14. Western blot analysis, as presented in Figure 6B, further verified this reduction at the protein level. After treatment with 50 and 100 nM Ang II, the OX2R protein levels decreased to 0.74 ± 0.072 and 0.51 ± 0.055, respectively. Taken together, these results confirm that Ang II suppresses OX2R expression at both the transcriptional and translational levels.

### 3.7 Orexin B Ameliorates Monocyte Adhesion to HBMVECs and Reduces SP‐1 Expression

**FIGURE 7:**
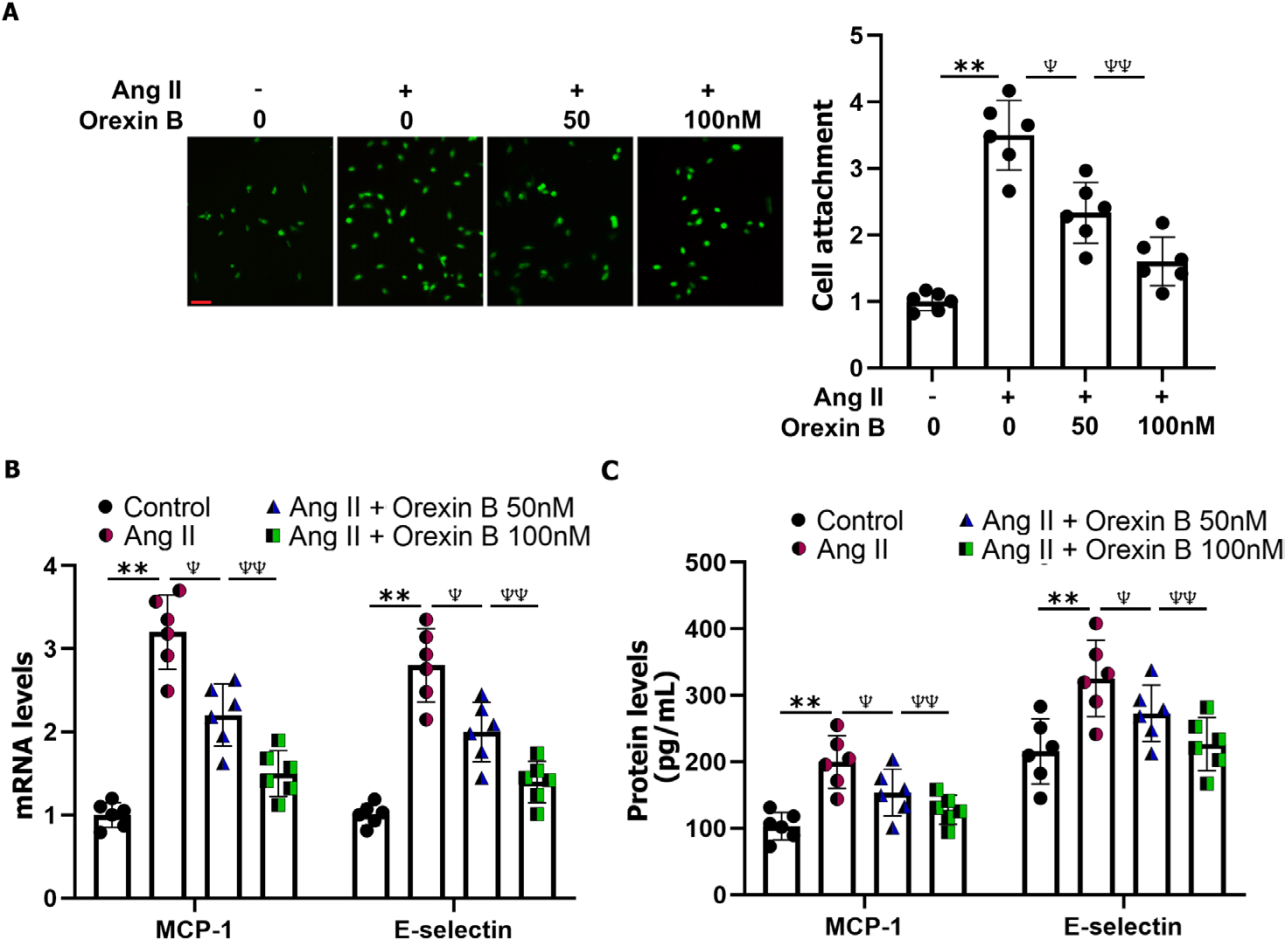
Orexin B ameliorates THP‐1 cell attachment to HBMVECs. (A) Calcein‐AM staining showed Ang II (100 nM)‐induced THP‐1 cell attachment to HBMVECs was reduced by Orexin B (50, 100 nM). Scale bar, 100 μm. (B) mRNA levels of *MCP‐1* and *E‐selectin* in HBMVECs were upregulated by Ang II and downregulated by Orexin B. (C) ELISA‐measured protein levels mirrored these changes. Data are mean ± SD. *n* = 6/group. **, *p* < 0.01 vs. Control group; Ψ, ΨΨ, *p* < 0.05, 0.01 vs. Ang II group (two‐way ANOVA with Tukey's test).

**FIGURE 8:**
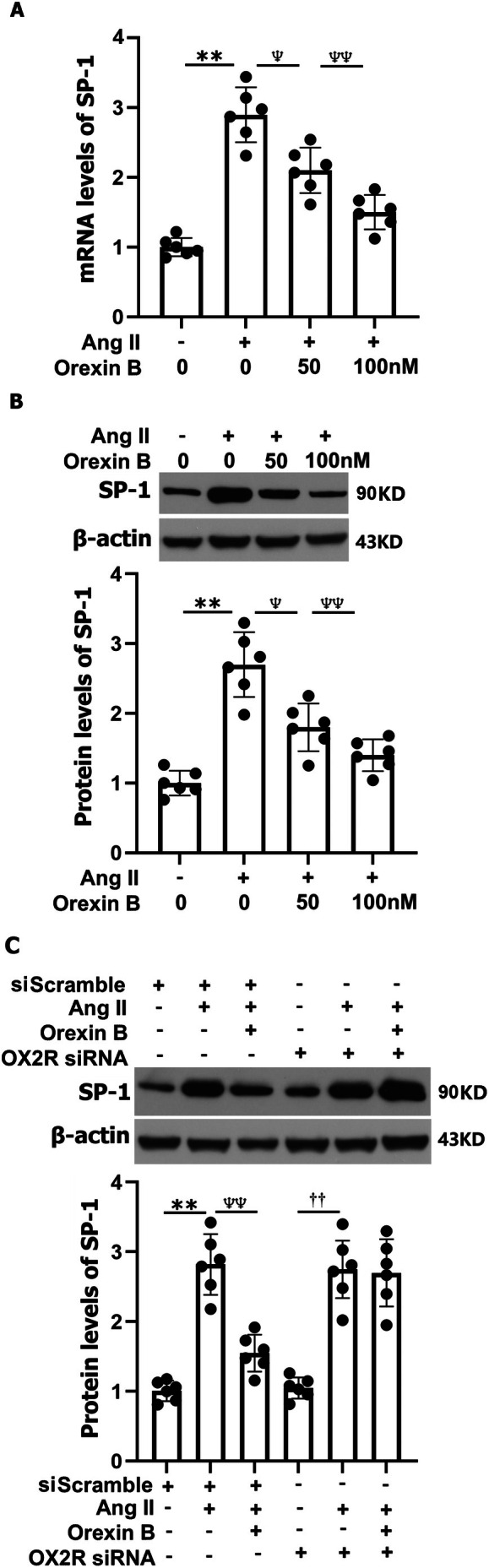
Orexin B reduces SP‐1 expression in Ang II‐stimulated HBMVECs. (A) Real‐time PCR showed Ang II (100 nM) increased *SP‐1* mRNA, which was reduced by Orexin B (50, 100 nM). (B) Western blot analysis confirmed reduced SP‐1 protein levels with Orexin B treatment. (C) siRNA‐mediated knockdown of OX2R abolished the ability of Orexin B to suppress Ang II‐induced SP‐1 protein upregulation. *n* = 6/group. **, *p* < 0.01 vs. Control group; Ψ, ΨΨ, *p* < 0.05, 0.01 vs. Ang II group; ††, vs. OX2R siRNA (two‐way ANOVA with Tukey's test).

Next, we explored the influence of Orexin B on the adhesion of monocytes to HBMVECs, a pivotal step within the inflammatory cascade. As shown in Figure 7A, the application of Ang II at a concentration of 100 nM boosted the adherence of THP‐1 human monocytic cells to HBMVECs. In contrast, treatment with Orexin B at 50 and 100 nM substantially lessened this adherence. Figure 7B,C displayed the mRNA and protein levels of MCP‐1 and E‐selectin in HBMVECs. Ang II treatment led to an elevation in the expression of MCP‐1 and E‐selectin, yet Orexin B treatment reverted this upward trend. Furthermore, Figure 8A,B indicated that Orexin B treatment decreased the expression of SP‐1, a transcription factor linked to pro‐inflammatory signaling, in HBMVECs that were stimulated by Ang II. Furthermore, siRNA‐mediated knockdown of OX2R in HBMVECs abolished the ability of Orexin B to suppress Ang II‐induced SP‐1 protein upregulation (Figure 8C), confirming that Orexin B regulates SP‐1 specifically through OX2R. These outcomes suggest that Orexin B inhibits the adhesion of monocytes to HBMVECs and dampens SP‐1‐mediated pro‐inflammatory signaling.

### 3.8 Overexpression of SP‐1 Abolishes the Inhibitory Effects of Orexin B

**FIGURE 9:**
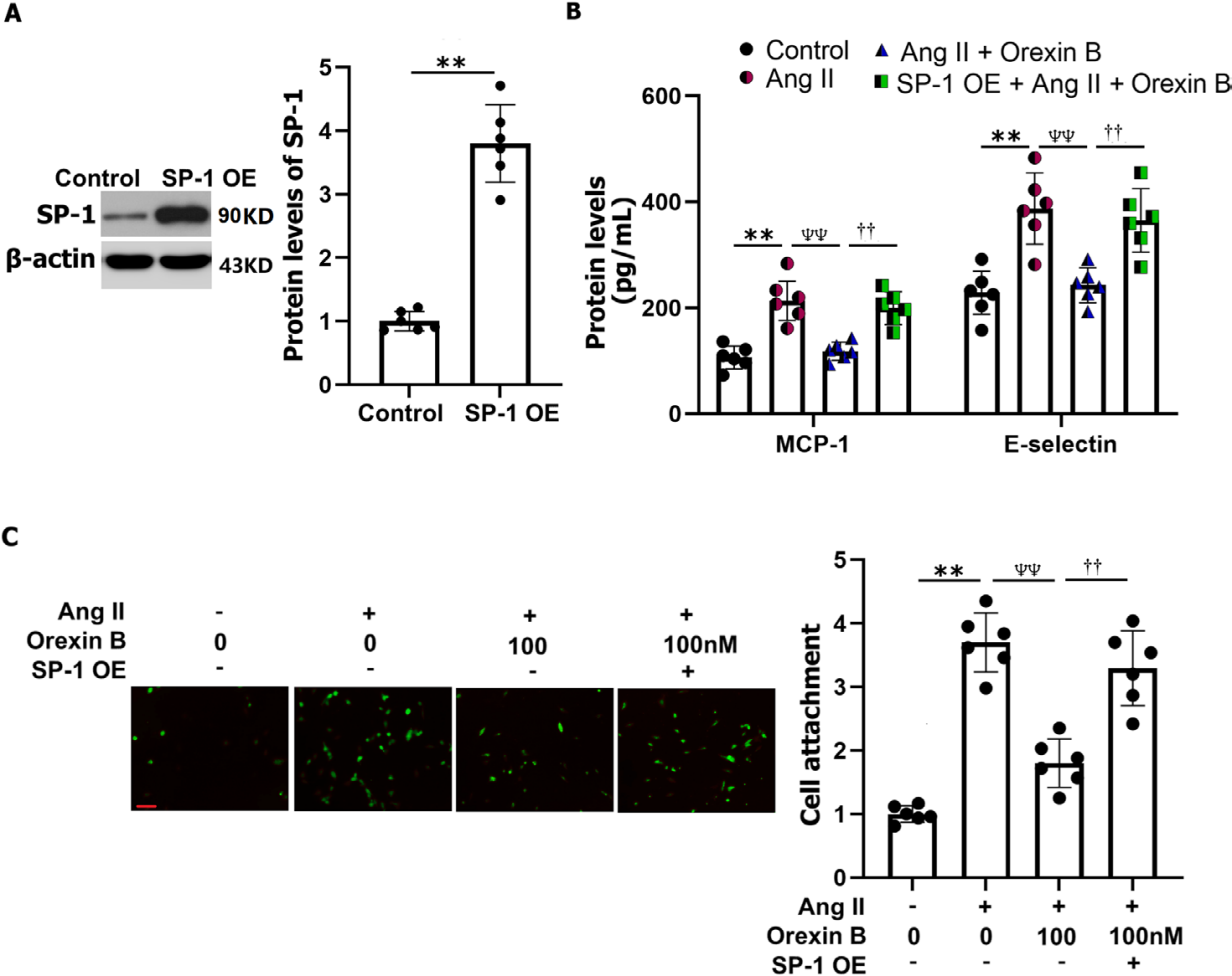
SP‐1 overexpression abolishes Orexin B's inhibitory effects. (A) Western blot confirmed successful SP‐1 overexpression in transduced cells (Unpaired two‐tailed *t*‐test). (B) ELISA showed SP‐1 overexpression abolished Orexin B's inhibitory effect on Ang II‐induced MCP‐1 and E‐selectin protein levels. (C) Calcein‐AM assays demonstrated that SP‐1 overexpression negated Orexin B's reduction in THP‐1 cell attachment. Representative fluorescence images are shown as insets. Scale bar, 100 μm. Data are mean ± SD. *n* = 6/group. **, *p* < 0.01 vs. Control group; ΨΨ, *p* < 0.01 vs. Ang II group; ††, *p* < 0.01 vs. Ang II + Orexin B group (two‐way ANOVA with Tukey's test).

To further confirm the role of SP‐1 in the protective effect of Orexin B, we overexpressed SP‐1 in HBMVECs using lentiviral vectors. Figure 9A shows the successful overexpression of SP‐1 by western blot analysis. Figure 9B presents that overexpression of SP‐1 abolished the inhibitory effect of Orexin B on the protein levels of MCP‐1 and E‐selectin in HBMVECs stimulated with Ang II. Figure 9C revealed that the number of attached THP‐1 cells to HBMVECs was significantly increased in the group with SP‐1 overexpression and Ang II treatment, even in the presence of Orexin B, indicating that SP‐1 overexpression negated the protective effects of Orexin B.

In conclusion, our results demonstrate that serum Orexin B levels are reduced in CA patients and animal models, and the Orexin B/OX2R system is dysregulated in CAs. Orexin B administration effectively attenuates CA formation through anti‐inflammatory mechanisms, including the inhibition of key inflammatory mediators, macrophage infiltration, and adhesion molecule expression. The protective effect of Orexin B is mediated through the OX2R and is associated with the inhibition of SP‐1‐mediated pro‐inflammatory signaling. These findings identify the Orexin B/OX2R axis as a potential therapeutic target for CAs.

## 4 Discussion

A key finding of our study is the identification of the Orexin B/OX2R axis as a potential therapeutic target for CAs. This is the first study to thoroughly examine the role of Orexin B in CA pathogenesis. By showing that Orexin B administration reduces CA formation in wild‐type mice but not in OX2R knockout mice, we establish a link between the Orexin B/OX2R system and CA development. Another important discovery is the mechanism behind Orexin B's protective effects. We found that Orexin B treatment decreases the expression of SP‐1, a transcription factor central to pro‐inflammatory signaling. Overexpression of SP‐1 abolishes the protective effects of Orexin B, underscoring SP‐1's critical role in the anti‐inflammatory and vascular‐protective actions mediated by Orexin B. This new insight into the molecular pathway offers a fresh perspective on CA pathophysiology.

Previous research has established that CAs are complex vascular disorders with a multifactorial etiology, involving genetic, hemodynamic, and inflammatory factors [19]. Our study was consistent with the existing knowledge regarding the role of inflammation in CA pathogenesis. Inflammatory mediators such as IL‐6 and MMP‐9 have been previously reported to contribute to the degradation of the extracellular matrix in the aneurysm wall, leading to its weakening and aneurysm formation [20]. Our findings show that Orexin B administration effectively suppresses the expression of IL‐6 and MMP‐9 in wild‐type mice, suggesting that Orexin B may modulate the inflammatory process in CAs, which is consistent with the known role of inflammation in this disease.

Regarding the Orexin system, prior studies have mainly focused on its role in regulating wakefulness, energy homeostasis, and stress response [21]. However, emerging evidence has hinted at its potential involvement in vascular function [7]. Our study expands on this by demonstrating a significant reduction in serum Orexin B levels in both CA patients and animal models, along with downregulation of OX2R expression in the COW region. This dysregulation of the Orexin B/OX2R system in CAs is a novel finding that is consistent with the growing body of evidence suggesting the Orexin system's role in non‐traditional physiological functions, but it is a new aspect in the context of CAs.

There are few direct comparisons with previous studies specifically on the relationship between Orexin B and CAs due to the novelty of this research area. However, in the broader context of neuropeptides and vascular diseases, some studies have shown that neuropeptides can modulate vascular tone and inflammation [22]. Our results showing that Orexin B inhibits monocyte adhesion to HBMVECs and reduces SP‐1‐mediated pro‐inflammatory signaling are in line with the general concept that neuropeptides can influence vascular‐inflammatory processes.

Orexin B's ability to inhibit macrophage infiltration and the expression of adhesion molecules such as MCP‐1 and E‐selectin provides insights into how it modulates the inflammatory microenvironment in the aneurysm wall. Macrophage infiltration is a characteristic feature of CAs, and the recruitment of macrophages is facilitated by adhesion molecules. By reducing these factors, Orexin B may prevent the chronic inflammation that contributes to aneurysm growth and rupture [23].

The identification of SP‐1 as a downstream target of Orexin B in the context of CAs is a major advancement. SP‐1 is involved in regulating genes related to inflammation and extracellular matrix remodeling. The negative regulation of SP‐1 by Orexin B may disrupt the pro‐inflammatory signaling cascade, leading to a decrease in the expression of genes that promote aneurysm development. This is supported by studies showing that GPCR signaling, such as that initiated by OX2R, can regulate transcription factors like SP‐1 [24, 25, 26, 27]. This finding not only elucidates the mechanism of Orexin B's action but also provides a potential new target for therapeutic intervention [28].

The significant reduction in serum Orexin B levels in CA patients and animal models is an important finding. It suggests that Orexin B may be a potential biomarker for CAs. Monitoring serum Orexin B levels could potentially help in the early detection of CAs or in predicting the risk of aneurysm rupture. In addition, it indicates that restoring Orexin B levels or enhancing its function may be a viable therapeutic strategy [29].

The observation that Orexin B administration attenuates CA formation in wild‐type mice but not in OX2R knockout animals emphasizes the importance of the OX2R in mediating the protective effects of Orexin B. This finding validates the role of the Orexin B/OX2R axis in CA development and suggests that targeting OX2R could be an effective way to modulate the action of Orexin B in the treatment of CAs [30].

The suppression of key inflammatory mediators (IL‐6 and MMP‐9) by Orexin B is significant, as it provides a direct link between Orexin B and the inflammatory processes involved in CA pathogenesis. Inflammation is a major driver of aneurysm growth and rupture, and by inhibiting these mediators, Orexin B may slow down or prevent the progression of CAs [31].

Orexin B's ability to diminish macrophage infiltration and downregulate adhesion molecules such as MCP‐1 and E‐selectin is also a pivotal mechanism. Macrophages are central drivers of the inflammatory cascade in CAs, and adhesion molecules act as critical mediators of their recruitment to the vulnerable aneurysm wall. By blunting these processes, Orexin B may disrupt the inflammatory feedback loop and safeguard the structural integrity of the aneurysm wall, thereby mitigating pathological vascular remodeling [32].

Finally, the finding that Orexin B reduces SP‐1 expression and that SP‐1 overexpression abolishes the protective effects of Orexin B highlights the importance of SP‐1 in the pathogenesis of CAs. This provides a new molecular target for developing therapies that can either inhibit SP‐1 or enhance the inhibitory effect of Orexin B on SP‐1 [33].

A limitation is our reliance on a single mouse model for in vivo studies. While widely used, it may not fully capture the complexity of human CAs, which involve diverse etiologies and mechanisms. Future work should incorporate multiple animal models or ex vivo human tissue to validate findings. Additionally, our human cohort included 38 patients and 43 controls, a sample size small to definitively establish serum Orexin B as a biomarker; larger clinical studies are needed. In vitro experiments using simplified cell cultures, overlooking the intricate cell interactions within the aneurysm microenvironment, require more sophisticated models mimicking in vivo conditions. Lastly, the study did not assess the long‐term effects of Orexin B treatment, leaving it unclear whether short‐term benefits translate to preventing rupture or improving outcomes. Furthermore, our model primarily evaluates aneurysm formation and growth; the incidence of spontaneous rupture in this model during our observation period is low and variable. Therefore, the effect of Orexin B on aneurysm rupture risk remains to be investigated in models specifically designed to study rupture.

Moving forward, our research should explore upstream regulators of the Orexin B/OX2R system to uncover new pathogenic insights and therapeutic targets. Developing OX2R‐specific agonists/antagonists or gene therapies targeting this axis or SP‐1 could offer novel treatment strategies. Investigating interactions between Orexin B and pathways like the renin‐angiotensin system, critical for vascular remodeling, will deepen understanding of CA mechanisms. Finally, translational efforts, including clinical trials evaluating Orexin B‐based therapies, are vital to realizing the therapeutic potential of this axis.

## 5 Conclusion

Our research has pinpointed the Orexin B/OX2R axis as a prospective therapeutic avenue for CA. We've demonstrated that the levels of Orexin B are decreased in both CA patients and animal models. Moreover, the administration of Orexin B can impede the formation of CA. It achieves this by engaging anti‐inflammatory processes and curbing the pro‐inflammatory signaling mediated by SP‐1. Nonetheless, additional research is required to comprehensively grasp the function of the Orexin B/OX2R axis in CA. Based on the findings from this study, we need to develop effective treatment strategies that can make full use of the potential of this axis.

## Funding

Dongguan Science and Technology of Social Development Program (No. 20231800935822); Talent Development Foundation of The First Dongguan Affiliated Hospital of Guangdong Medical University (No. GCC2023007); Guangdong Medical Research Fund project (No. A2023438).

## Ethics Statement

Ethical oversight for this research involving human samples was managed by the Medical Ethics Committee at the First Dongguan Affiliated Hospital at Guangdong Medical University (Approval No. NTZ5686). Procedures involving animals were reviewed and authorized by the Animal Ethics Oversight Board of the First Dongguan Affiliated Hospital at Guangdong Medical University (Approval No. NTC5701).

## Consent

All the authors agreed to publish this article.

## Conflicts of Interest

The authors declare no conflicts of interest.

## Supporting information


**Figure S1:** H&E staining of the COW region. Representative H&E images showing reduced vascular wall layering, decreased smooth muscle cell count, fragmentation of elastic fibers, and intimal disruption in the aneurysm dome of CA mice (B), compared with the control group (A). Red arrows indicate endothelial cells, and black arrows indicate smooth muscle cells.

**Figure S2:** Baseline characterization of *OX2R* ^−/−^ mice. (A) Serum IL‐6 levels and (B) COW MMP‐9 protein levels in WT and *OX2R* ^−/−^ mice under sham or CA conditions. No significant differences were observed between genotypes. **p < 0.01 vs. WT group; ††*p* < 0.01 vs. *OX2R* ^−/−^ group. Data are mean ± SD; *n* = 6 mice/group (two‐way ANOVA with Tukey's test).

## Data Availability

The data that support the findings of this study are available from the corresponding author upon reasonable request.
