# Supplementary material for: Orexin B Reduces Cerebral Aneurysms Through Inhibition of SP‐1
Source: CNS Neurosci Ther. 2026 Jun 8;32(6):e70958. doi: 10.1002/cns.70958 (PMC13245276; doi:10.1002/cns.70958)
Supplement: Supplementary file 1 — Figure S1: H&E staining of the COW region. Representative H&E images showing reduced vascular wall layering, decreased smooth muscle cell count, fragmentation of elastic fibers, and intimal disruption in the aneurysm dome of CA mice (B), compared with the control group (A). Red arrows indicate endothelial cells, and black arrows indicate smooth muscle cells. [file CNS-32-e70958-s002.docx]

**
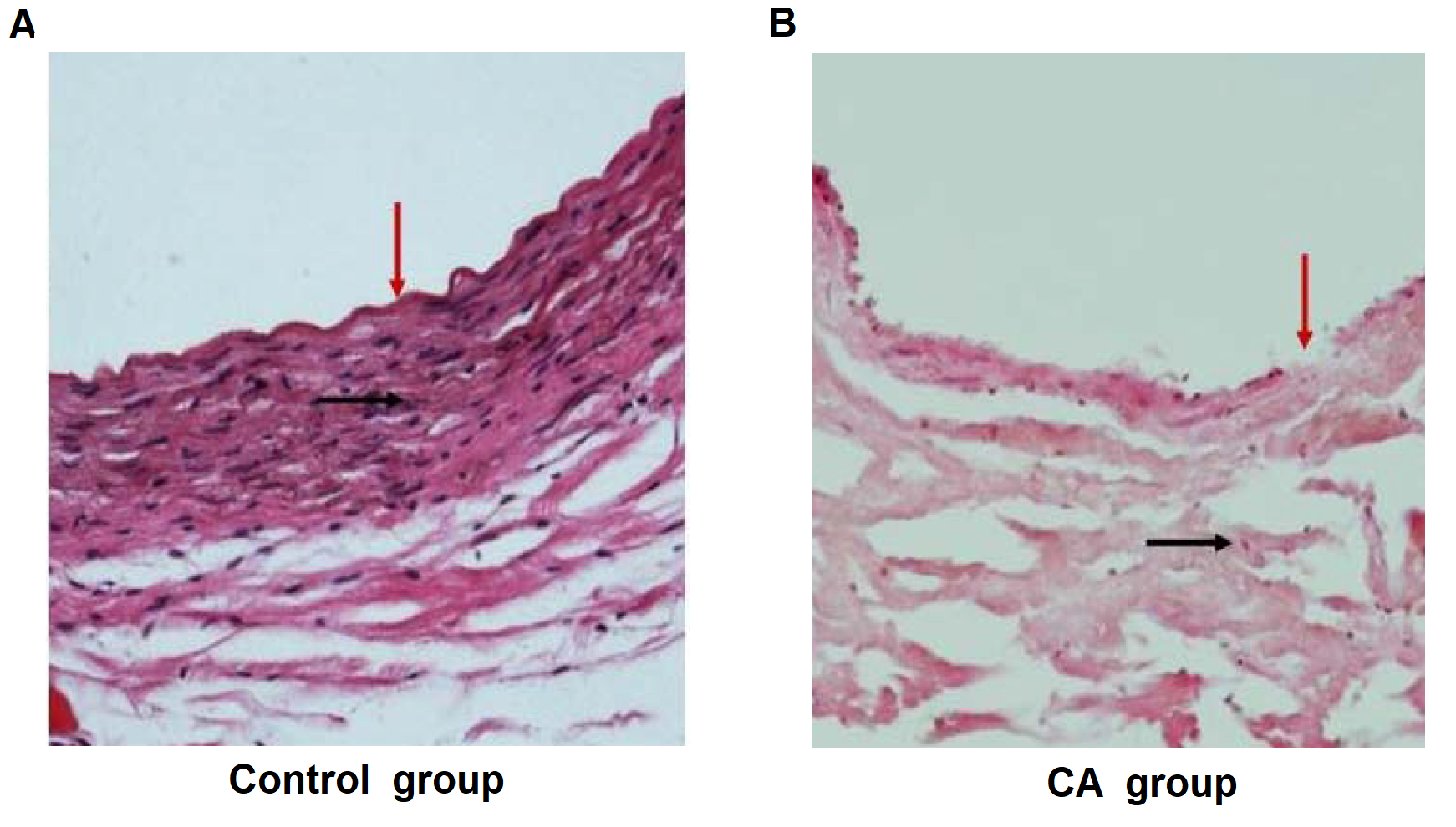
**

**Supplementary Figure S1:** Supplementary Figure S1: H&E staining of the COW region. Representative H&E images showing reduced vascular wall layering, decreased smooth muscle cell count, fragmentation of elastic fibers, and intimal disruption in the aneurysm dome of CA mice (B), compared with the control group (A). Red arrows indicate endothelial cells, and black arrows indicate smooth muscle cells.
