# Supplementary material for: Orexin B Reduces Cerebral Aneurysms Through Inhibition of SP‐1
Source: CNS Neurosci Ther. 2026 Jun 8;32(6):e70958. doi: 10.1002/cns.70958 (PMC13245276; doi:10.1002/cns.70958)
Supplement: Supplementary file 2 — Figure S2: Baseline characterization of OX2R −/− mice. (A) Serum IL‐6 levels and (B) COW MMP‐9 protein levels in WT and OX2R −/− mice under sham or CA conditions. No significant differences were observed between genotypes. **p < 0.01 vs. WT group; ††p < 0.01 vs. OX2R −/− group. Data are mean ± SD; n = 6 mice/group (two‐way ANOVA with Tukey's test). [file CNS-32-e70958-s001.docx]

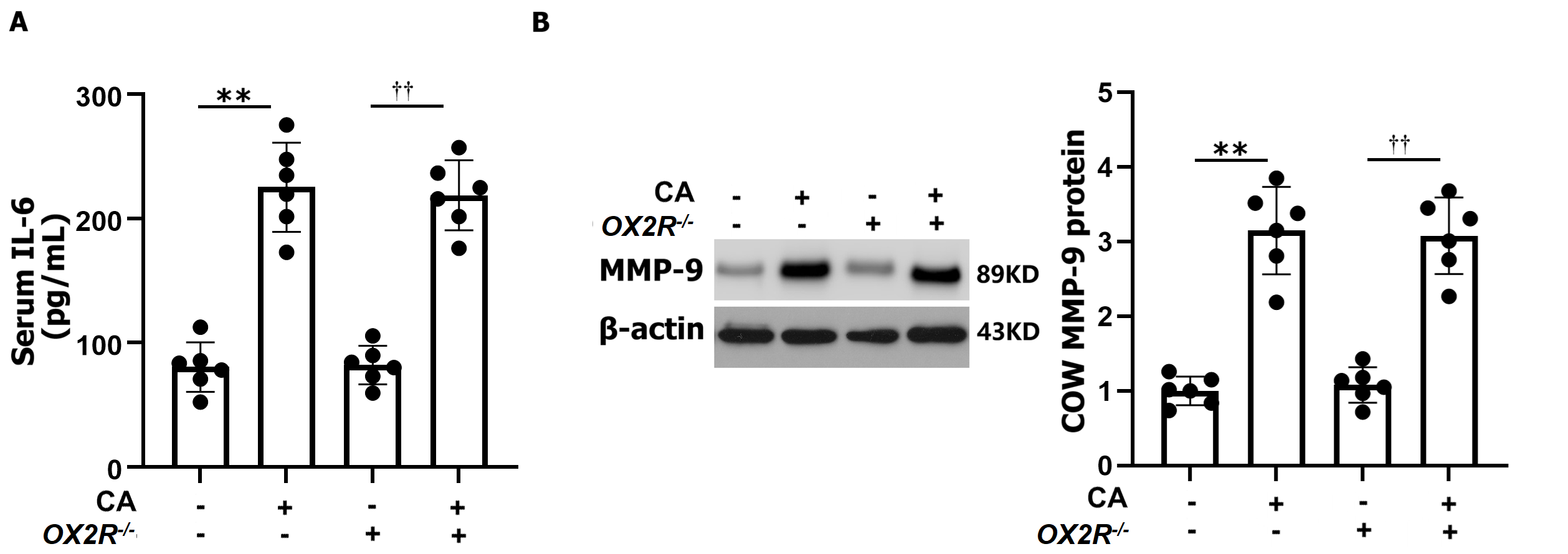


**Supplementary Figure S2:** Baseline characterization of *OX2R^-/-^* mice. (A) Serum IL-6 levels and (B) COW MMP-9 protein levels in WT and *OX2R^-/-^* mice under sham or CA conditions. No significant differences were observed between genotypes. **, P<0.01 vs WT group; ††, P<0.01 vs. *OX2R^-/-^* group. Data are mean ± SD; n=6 mice/group. (two-way ANOVA with Tukey's test).
